# Simulation based design tool for radial impellers of centrifugal pumps

**DOI:** 10.1038/s41598-024-62808-3

**Published:** 2024-06-06

**Authors:** Ahmed Farid Saad Ayad Hassan, Anwer Hashish, Sherif Saleh

**Affiliations:** Military Technical College, Cairo, Egypt

**Keywords:** Energy infrastructure, Mechanical engineering

## Abstract

Centrifugal water pumps account for more than 20% of energy use all over the world. Even a little increase in centrifugal pump operating efficiency may result in billions of dollars in savings each year and help reduce carbon dioxide emissions. This study creates a design tool that integrates one-dimensional equations and computational fluid dynamics (CFD) simulations to design centrifugal pump impellers. This examines different combinations of design factors by utilizing both the powerful numerical tools that allow the modeling of numerous scenarios and the large number of empirical equations and coefficients that represent the significant knowledge accumulated in this field. The design tool runs through a series of equations to select the beginning candidates for the different impeller design variables. After that, a design space is established by generating upper and lower bounds for each design variable. The CFD tool executes numerous scenarios inside the design space in order to find the best candidates with the maximum operating efficiency and meet the design objectives. A low specific speed centrifugal pump with a semi-open impeller is used as a case study. The design tool is successful in designing it, fulfilling the design requirement, and increasing operational efficiency when compared to a simple single-arc comparable impeller.

## Introduction

Centrifugal pumps recorded the highest revenue in the water pump market by $30.56 billion in 2021. Due to the global warming problem and the increase in the world population, the centrifugal pump market is expected to increase to $45.56 billion by 2028, presenting a Compound Annual Growth Rate (CAGR) of 5.9% in this period^1^. Water pumps are responsible for over 20% of world energy consumption^2,3^. Therefore, any enhancement in the centrifugal pump operating efficiency, even at a slight rate, saves billions of USD annually and contributes to carbon dioxide emission reduction^3–5^.

Centrifugal pumps are designed using empirical data to predict their performance and efficiency as a result of their complex flow phenomena. A flow channel and blade design are then based on experience and coefficients derived from test results^6^. The development of numerical algorithms that can reasonably solve the 3-dimensional Navier-Stokes equations with complex components has been aided by the advent of relatively cheap computers with high processing speeds. As a result, the pump sector also uses Computational Fluid Dynamics (CFD) tools to optimize hydraulic components, improve the accuracy of performance prediction, and consequently save testing costs^7^.

In this work, a design tool that combines one-dimensional and CFD design methods is created. This makes the use of both the extensive knowledge amassed in this area, represented by the vast number of empirical equations and coefficients, as well as the potent numerical tools that enable the simulation of many scenarios to look at various combinations of design factors. To choose the initial candidates for the various impeller design variables, the design tool first executes a set of equations. Then, it creates upper and lower bounds for all design variables to establish a design space. In order to identify the best candidates with the highest operational efficiency and achieve the design goals, the CFD tool performs many situations inside the design space.

As a case study, a centrifugal pump with a semi-open impeller and a low specific speed is used. Compared to a basic single-arc similar impeller, the design tool is successful in designing it, meeting the design requirement, and improving operational efficiency.

## Design tool

**Figure 1:**
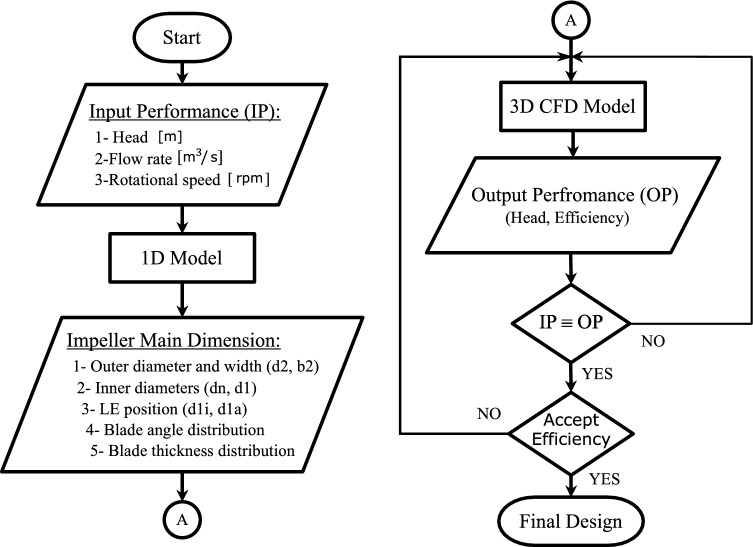
Semi-open impeller design tool flowchart.

The centrifugal impeller design tool consists of two main steps as shown in Fig. 1. First, it runs a 1D model to generate the primary dimensions of the impeller. Second, it performs a 3D CFD analysis to evaluate the performance of the selected model from the previous step. the design tool repeats the second step to fine-tune the model and achieve accurately the required pump performance.

### One dimensional model

The 1D model consists of a group of equations gathered from different references and the own experience. All these equations are combined and programmed using the open-source high-level programming language Python. The input data for the 1D model is the pump head $H_{opt} \mathrm {[m]}$, flow rate $Q_{opt} \mathrm {[m^3/s]}$, and the rotational speed $n \mathrm {[rpm]}$ at the best efficient point of the pump operation. The 1D model starts by calculating the specific speed $n_q$.

1 $$\begin{aligned} n_q = n \frac{\sqrt{Q_{opt}}}{H_{opt}^{0.75}} \end{aligned}$$

Then the model estimates the hydraulic $\eta _{h,opt}$ and total efficiency $\eta _{opt}$ using Eqs. (2–3), respectively.

2 $$\begin{aligned} \eta _{h,opt} = 1-0.055 {\left( \frac{Q_{Ref}}{Q}\right) }^{m_1}-0.2\left\{ 0.26-\log \frac{n_q}{25}\right\} ^2\left( \frac{Q_{Ref}}{Q}\right) ^{0.1} \end{aligned}$$

$$\begin{aligned} m_1&= 0.08 \ a \ \left( \frac{Q_{Ref}}{Q}\right) ^{0.15} \ \left( \frac{45}{n_q}\right) ^{0.06} \\ a&= {\left\{ \begin{array}{ll} 1 \ &{} Q \le 1 m^3/s\\ 0.5, \ &{} Q > 1 m^3/s \end{array}\right. } \end{aligned}$$

where $Q_{Ref}$ is the reference flow rate and it equals 1 $\mathrm {m^3/s}$ for $$Q\le 0.005 \ $$ $\mathrm {m^3/s}$.

3 $$\begin{aligned} \eta _{opt} = 1-0.095 {\left( \frac{Q_{Ref}}{Q}\right) }^{m_2}-0.3\left\{ 0.35-\log \frac{n_q}{23}\right\} ^2\left( \frac{Q_{Ref}}{Q}\right) ^{0.05} \end{aligned}$$

$$\begin{aligned} m_2&= 0.1 \ a \ \left( \frac{Q_{Ref}}{Q}\right) ^{0.15} \ \left( \frac{45}{n_q}\right) ^{0.06} \end{aligned}$$

After estimating the hydraulic and total efficiency, the volumetric efficiency $\eta _v$ is calculated from the following equation:

4 $$\begin{aligned} \eta _{v,opt} = \frac{\eta _{opt}}{\eta _h \times \eta _m} \end{aligned}$$

where $\eta _m$ is the mechanical efficiency which assumed to be 0.95 in the 1D model. Then, the model calculate the impeller shaft diameter $d_w$ using the following equation:

5 $$\begin{aligned} d_{w} = 3.65 \times SF \times (\frac{P_{max}}{n \ \tau _{al}}) \end{aligned}$$

where $\tau _{al}$ is the allowable shear strength of the shaft material $\mathrm {[N/m^2]}$, and $P_{max}$ is the maximum expected power and is calculated from the following equation:

6 $$\begin{aligned} P_{max} = {\left\{ \begin{array}{ll} 1.3 \times P_{opt} \ &{} n_q\le 100\\ 2 \times P_{opt} \ &{} 100< n_q < 200\\ 3 \times P_{opt}, \ &{} n_q \ge 200 \end{array}\right. } \end{aligned}$$

where $P_{opt}$ is the shaft power consumption at BEP which is calculated from the following equation:

7 $$\begin{aligned} P_{opt} = \rho \times g \times H_{opt} \times Q_{opt} / \eta _{opt} \end{aligned}$$

**Figure 2:**
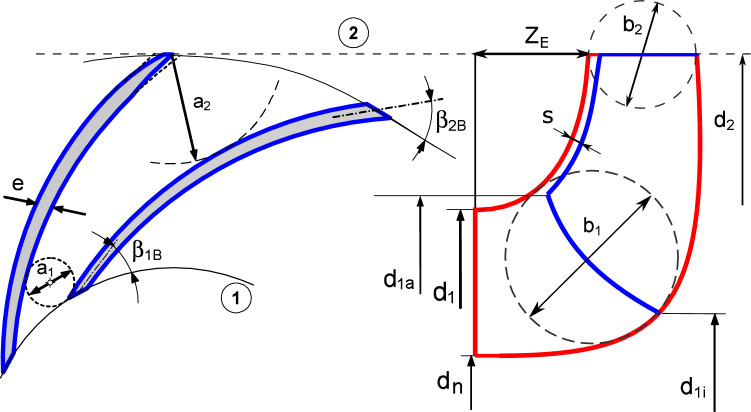
Semi-open impeller main dimensions.

It is frequently necessary to increase the shaft diameter above the minimum that has been calculated based on the torque only by 20:40% based on the shaft size. Then the model starts calculate the impeller blade number $z_{La}$ and main dimensions: Impeller outlet, inlet, hub diameters $(d_2, d_1, d_n)$, Leading Edge (LE) hub and shroud diameters $(d_{1i}, d_{1a}$, impeller outlet width $(b_2)$, blade extension $(Z_{E})$, blade inlet and outlet angles $(\beta _{1B}, \beta _{2B})$, and the blade thickness (*e*). The main dimensions of the impeller are represented in Fig. 2.

**Figure 3:**
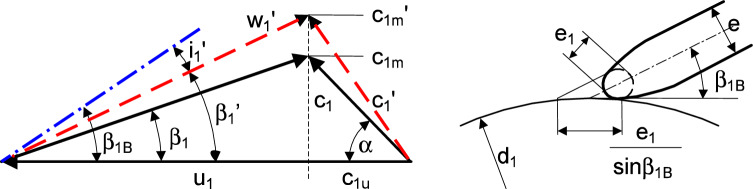
Impeller inlet section and velocity triangle.

- *Outlet diameter* $(d_2)$: The impeller outlet diameter is calculated using the definition of the head coefficient at the BEP $\psi _{opt}$ as follows:
  8 $$\begin{aligned} d_2 = \frac{60}{\pi \ n} \times \sqrt{\frac{2 g H_{opt}}{\psi _{opt}}} \end{aligned}$$
  The head coefficient at the BEP is calculated using the following analytical equation as a function of the specific speed as follows:
  9 $$\begin{aligned} \psi _{opt} = 1.21 \ e^{-0.77 n_q / n_{q,Ref}} \end{aligned}$$
  where $n_{q,Ref}$ is the reference specific speed equal 100.
- *Blade number* $(z_{La})$: Selecting the blade number is an optimization between the flow slip, the friction losses, and the dynamic behavior of the impeller. The 1D model set the blade number initially to 6 blades until it will optimized during the 3D Model.
- *Hub diameter* $(d_{n})$: The hub diameter is calculated based on the shaft diameter from the following equation:
  10 $$\begin{aligned} d_n = R_n \times d_w \end{aligned}$$
  where $R_n$ is the hub-to-shaft diameter ratio. This value is set up to 1.5 as a default value but will be chosen as a design parameter in the 3D model.
- *Inlet diameter* $(d_{1})$: Selecting the inlet diameter within this model is based on achieving minimum relative velocity at the impeller inlet. This design tends to minimize leakage, friction, and shock losses. It is recommended when the available Net Positive Suction Head $(NPSH_A)$ is sufficiently high so that cavitation is avoided. There for the inlet diameter is calculated from the following relation:
  11 $$\begin{aligned} d_1 = d_2 \ f_{d1} \sqrt{\left( \frac{d_n}{d_2}\right) ^2+1.48 \times 10^{-3} \psi _{opt} \frac{n_q^{1.33}}{\eta _v \delta _r}} \end{aligned}$$
  where $\delta _r = 1- c_{1m}/(u_{1m} \ \tan \alpha _1), c_{1m}$ and $u_{1m}$ the absolute and base velocity at the mean inlet streamline, $\alpha _1$ is the absolute inflow angle. within the 1D model, the flow is assumed to enter the impeller radially therefore $\alpha _1=90$ and $\delta _r=1$. $f_{d1}=1.15 \ \text {to} \ 1.05$ falling from $n_q= 15 \ \text {to} \ 40$ for normal radial impeller.
- *LE diameter at hub and shroud* $(d_{1i}, d_{1a})$: The Leading Edge (LE) is design as straight line between the hub and shroud. It starts on the hub line at diameter $(d_{1i})$ and ends on the shroud at diameter $(d_{1a})$ as shown in Fig. 2. The LE hub and shroud diameters $(d_{1i}, d_{1a})$ are calculated using the following equations:
  12 $$\begin{aligned} d_{1i}= & {} [f_{d1i} \ (d_1-d_n )]+d_n \end{aligned}$$
  13 $$\begin{aligned} d_{1a}= & {} (1+f_{d1a})*d_1 \end{aligned}$$
  where $f_{d1i}, f_{d1a}$ is a design coefficients initialized as 0.3 and 0.125 as a default value. These values will optimized based on the design objectives in the 3D CFD model.
- *Blade thickness* (*e*): Selecting the blade thickness is a compromisation between the castability and mechanical strength. To satisfy this objective the blade thickness is selected as:
  14 $$\begin{aligned} e/d_2 = 0.016 \ \text {to} \ 0.022 \end{aligned}$$
  The design model uses $e/d_2=0.019$ by default and for the casting consideration if the e is lower than 3 the model set it by default as 3 mm.
- *Blade inlet angle* $(\beta _{1B})$: After determining the blade inlet diameter, the inlet velocity triangle as shown in Fig. 3 is determined as follows:
  $$\begin{aligned} u_1&= \pi d_1 n /60 \\ A_1&= \frac{\pi }{4} (d_1^2-d_n^2) \\ Q_{La}&= \frac{Q_{opt}}{\eta _{v,opt}} \\ c_{1m}&= \frac{Q_{La}}{A_1} \\ c_{1u}&= \frac{c_{1m}}{\tan \alpha _1} \\ w_{1}&= \sqrt{c_{1m^2}+(u_1-c_{1u})^2} \end{aligned}$$
  After determining the inlet velocity triangle, the design model calculate the inlet blade angle as follows:
  15 $$\begin{aligned} \beta _1 = \arctan \frac{c_{1m}}{u_1-c_{1u}} \end{aligned}$$
  Inlet blade thickness causes a inlet blockage and increases the meridional velocity at the impeller inlet as shown in Fig. 3. Therefore, the inlet blade angle with blockage is calculated using the following equation:
  16 $$\begin{aligned} \beta _1^\prime = \arctan \frac{c_{1m} \ \tau _1}{u_1-c_{1u}} \end{aligned}$$
  where $\tau _1$ is the blade blockage at impeller inlet due to the blade thickness.
  17 $$\begin{aligned} \tau _1= \left\{ 1-\frac{z_{La} \ e_1}{\pi d_1 \sin \beta _{1B} \sin \lambda _{La}}\right\} ^{-1} \end{aligned}$$
  where $\lambda _{La}$ is the angle between blades and the rotor side disks, $90^\circ$ as a default value in the model. The flow rate of shockless entry is thus commonly slightly above the best efficiency point. Therefore, the blade inlet angle increases slightly by adding the an incidence $i_1$
  18 $$\begin{aligned} \beta _{1B}= \beta _1^\prime + i_1 \end{aligned}$$
  where $i_1$ is in range from 0 to $4^\circ$ and is set up as $2^\circ$ within the design model.
- *Outlet width* $(b_2)$: A sufficiently wide impeller outlet width is desired to obtain a stable H-Q curve (especially at low $n_q$). However, the flow non-uniformity at the impeller outlet increases with the impeller outlet width. In addition, it should satisfy $b_2 < b_1$. Since this effects cannot be calculated theoretically, the outlet width is selected using the following empirical equation:
  19 $$\begin{aligned} b_2/d_2= 0.017+0.262 \left( \frac{n_q}{n_{q,Ref}}\right) -0.08 \left( \frac{n_q}{n_{q,Ref}}\right) ^2+0.0093 \left( \frac{n_q}{n_{q,Ref}}^3\right) \end{aligned}$$
- *Outlet blade angle* $(\beta _{2B})$:The blade outlet angle should be specified so that the pump head is achieved with the selected values of $d_2, b_2, \ \text {and} \ z_{La}$ which are determined previously. The blade outlet angle $\beta _{2B}$ which achieves this requirement can be calculated iteratively by calculating the respective slip factor $\gamma$ and the head as shown in Eqs. (20–21), respectively.
  20 $$\begin{aligned} \gamma = f_1 \left( 1-\frac{\sqrt{\sin \beta _{2B}}}{{z_{La}}^{0.7}}\right) \end{aligned}$$
  where $f_1$ is constant equal 0.98 for radial turbine, and $k_w=1$ for $d_{1m}/d_2 \le \varepsilon _{lim}$ else:
  $$\begin{aligned} k_w= 1-\left( \frac{\frac{d_{1m}}{d_2}-\varepsilon _{lim}}{1-\varepsilon _{lim}}\right) ^3 \end{aligned}$$
  where $d_{1m}$ is the impeller inlet mean diameter, and $\varepsilon _{lim}$ is calculated from the following equation:
  $$\begin{aligned} \varepsilon _{lim}= \exp \left\{ -\frac{8.16 \sin \beta _{2B}}{z_{La}}\right\} \end{aligned}$$
  21 $$\begin{aligned} H=\frac{\eta _h u_2^2}{g} \ \left\{ \gamma -\frac{Q_{La}}{A_2 u_2 \tan \beta _{2B}} \ \left[ \tau _2 + \frac{A_2 \frac{d_{1m}}{d_2} \tan \beta _{2B}}{A_1 \tan \alpha _1}\right] \right\} \end{aligned}$$
  where $\tau _2$ is the impeller outlet blockage due to the blade thickness and is calculated as follows:
  22 $$\begin{aligned} \tau _2= \left\{ 1-\frac{z_{La} \ e_2}{\pi d_2 \sin \beta _{2B} \sin \lambda _{La}}\right\} ^{-1} \end{aligned}$$
- *Axial extension* $(Z_E)$: The impeller axial extension is represented in figure and calculated as a function of the specific speed using the following equation:
  23 $$\begin{aligned} Z_E= (d_2-d_1) \left( \frac{n_q}{74}\right) ^{1.07} \end{aligned}$$

### CFD 3D model

**Figure 4:**
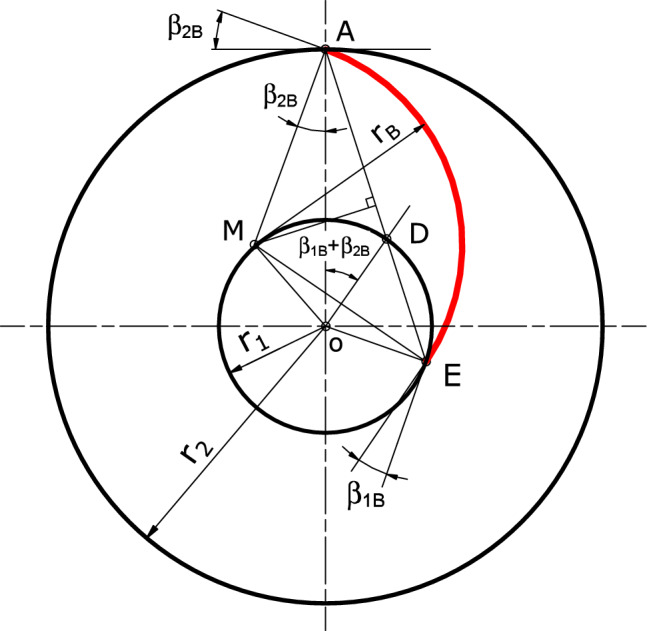
Single arc technique for backward blade construction.

This section discusses in detail the CFD 3D model. Starting from the outputs of the previous step, the CFD model first creates the 3D CAD of the impeller and CFD domain. To achieve this, the blade angles and thickness distribution along the meridional direction are required. In this design tool, the blade angle distribution (as a function of the impeller radius (*r*)) is based on the single arc technique as shown in the Fig. 4 and determined using Eq. (24).

24 $$\begin{aligned} \beta (r) = \cos ^{-1} \left\{ \frac{r_B^2+r^2-{\overline{MO}}^2}{2 \ r \ r_B}\right\} \end{aligned}$$

where $r_B$ and ${\overline{MO}}$ is blade radius and distance between impeller and blade center, respectively. they are determined from the following relations:

25 $$\begin{aligned} r_B= & {} \frac{r_2^2-r_1^2}{2[r_2 \cos \beta _{2B} - r_1 \cos \beta _{1B}]} \end{aligned}$$

26 $$\begin{aligned} {\overline{MO}}^2= & {} r_B^2 + r_2^2 - 2 r_B r_2 \cos \beta _{2B} \end{aligned}$$

This design model adjusts the thickness distribution initially as constant along the blade radius until it will be optimized after determining the static and dynamic loads.

**Figure 5:**
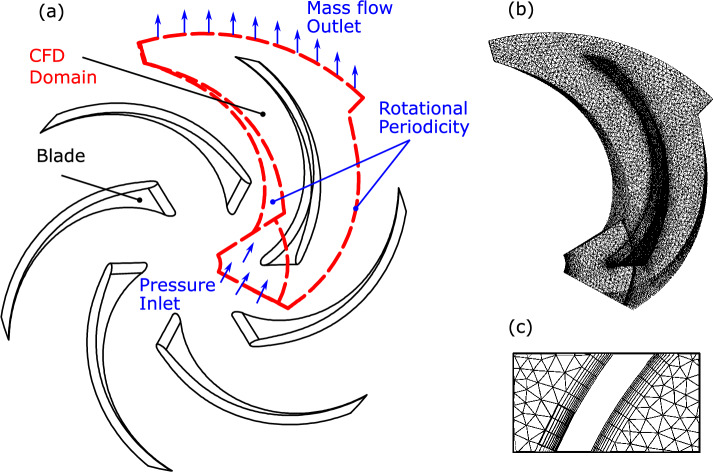
Impeller 3D model: (**a**) impeller CAD model and CFD sector domain (in dash lines) (**b**) sector domain mesh (**c**) blade inflation layers.

Using all previously determined geometrical data, the design model builds the impeller CAD model using ANSYS Design Modeler. It first creates the meridional plan, then the blade camber surface using the blade angle distribution then it constructs the blade using the blade thickness distribution. The next step is to create the CFD domain. This study is interested in general performance prediction therefore one blade sector model is constructed because it is a compromise between the solution accuracy and the design time. An instance of the constructed blade and the CFD on sector domain are presented in Fig. 5a.

After creating the CFD domain, the design tool uses ANSYS ICEM to mesh the CFD domain. It uses an unstructured mesh technique to generate the mesh to facilitate the full automation of the design tool as shown in in Fig. 5b,c. The domain mesh has 250 k elements to achieve a solution independent of the mesh size.

**Figure 6:**
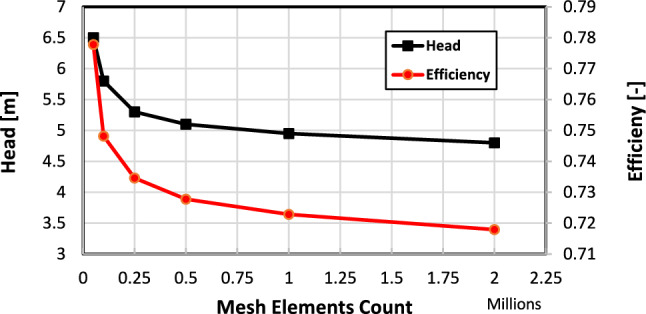
Mesh sensitivity analysis, Q = 3.5 L/s.

The domain mesh is selected with 250 k elements to achieve a solution independent of the mesh size. This mesh elements count is chosen according to the mesh sensitivity analysis as presented in Fig. 6. In the sensitivity analysis, the mesh refinement is done to keep the y+ the same (less than 1) while increasing the element count in stream-wise and span-wise directions. The y+ adjusted (less than 1) by using ten inflation prism layers with the first layer distance from the blade, hub, and shroud walls of 0.01 mm and a growth rate of 1.2 between layers. The convergence criterion is monitoring the pump head and hydraulic efficiency with the mesh element count. The results show that selecting 250 k elements achieved acceptable solution accuracy in a suitable computational time to allow comparing large number of designs to get the optimum one.

The mesh has 250 k elements for one impeller blade sector is found to be the minimum amount for sufficient local mesh refinement at required positions such as leading and trailing edges and provides sufficient prismatic boundary layer to achieve the required y+. The mesh is generated fully automatically with predefined mesh parameters for each design. This is the main reason behind using the unstructured grid technique. It allows fast and reliable mesh generation with predefined mesh refinements at named selected parts such as the leading and trailing edges and allows generating a prismatic boundary layer with predefined parameters.

## Experimental work and CFD validation

**Figure 7:**
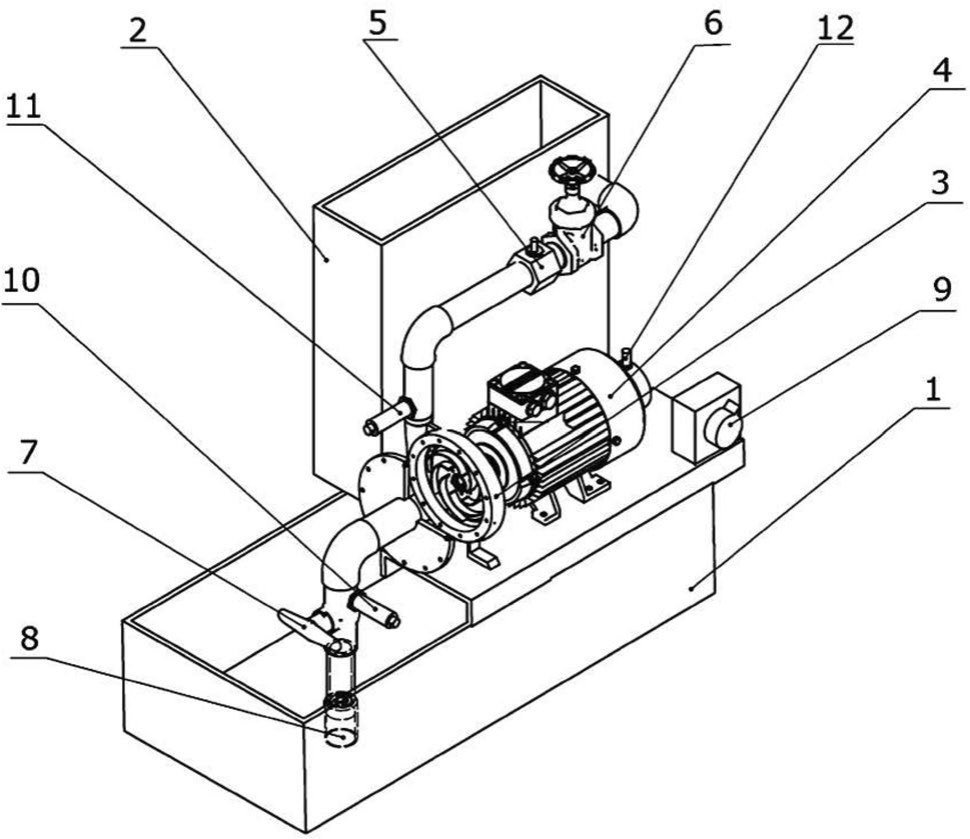
Centrifugal pump test rig: (1) sump tank, (2) volumetric measure tank, (3) centrifugal pump, (4) DC motor, (5) turbine flow meter, (6) delivery gate valve, (7) suction valve, (8) non-return valve, (9) speed control unit, (10) suction pressure sensor, (11) delivery pressure sensor, (12) electric tachometer.

A centrifugal pump featuring a semi-open impeller is tested experimentally using a centrifugal pump test rig at the mechanical power department at Military Technical College, Cairo. A schematic drawing of the test rig including its main components and measurement instruments is shown in Fig. 7.

**Figure 8:**
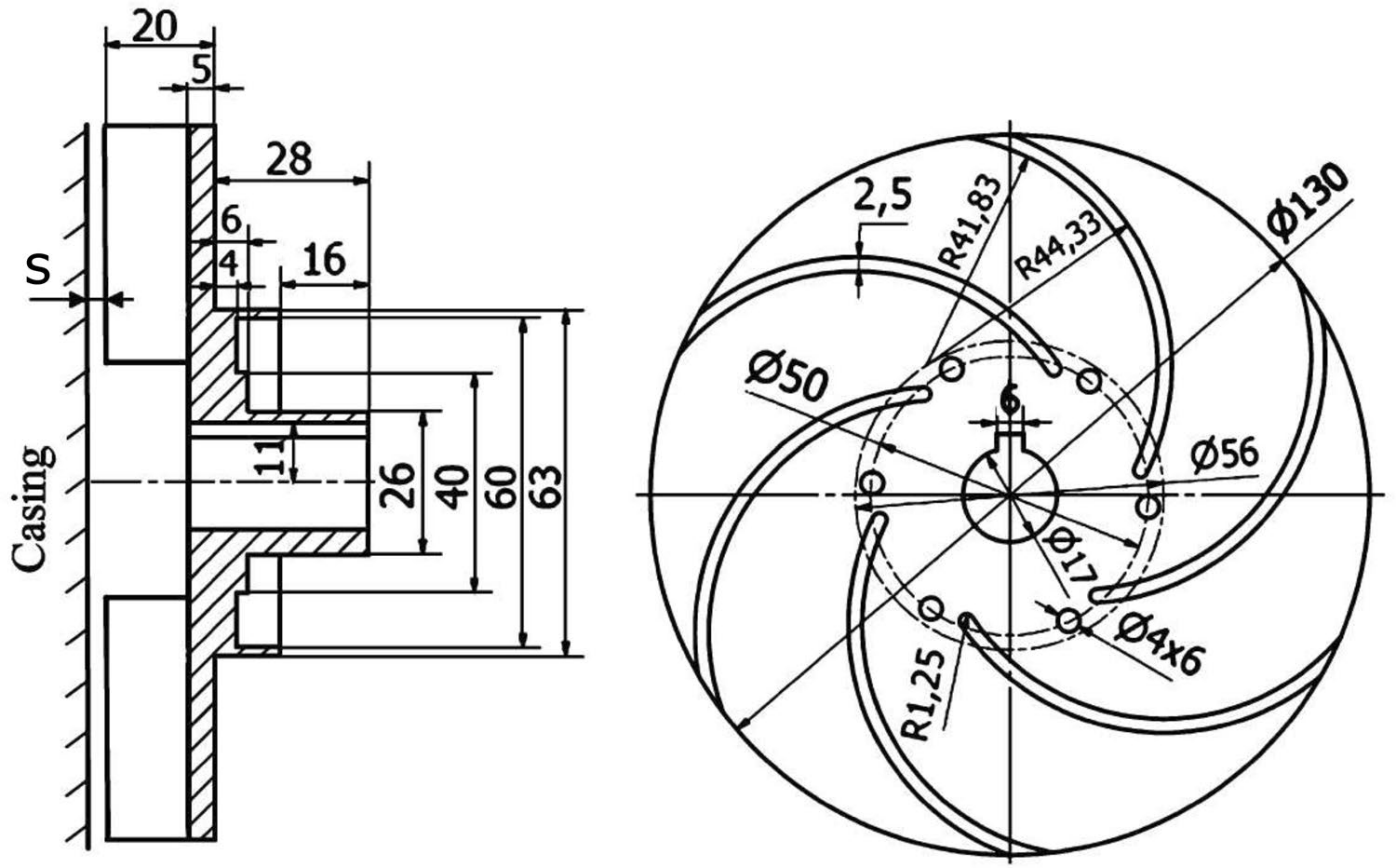
Impeller main dimensions (mm).

A centrifugal pump with semi-open impeller (Baseline model) is tested experimentally at a rotational speed of 1500 rpm and side clearance $s=1$ mm. A gate valve on the discharge pipe is used to control the volumetric flow rate and apply different operating points. Absolute pressure transducers (HBM P6A) are used at the pump suction and delivery pipes to accurately measure the inlet and outlet pressures. OMEGA FTB-109 turbine meter is used to measure the volumetric flow rate. A detailed explanation of the used instrumentation including (model, accuracy, and calibration) is mentioned in a previous publication^8,9^. The impeller has six backward blades and is designed based on a single arc technique with an inlet blade angle $\beta _1=35^\circ$ and outlet blade angle $\beta _2=20^\circ$. The impeller main dimensions are plotted in Fig. 8.

**Figure 9:**
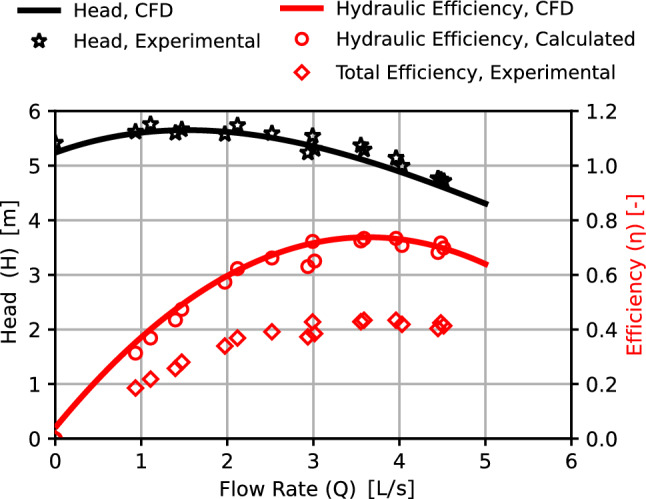
Validation of CFD model for impeller side clearance s = 1 mm.

For a total of 10 repetitions, the measuring procedure was done five times for a particular flow rate in a rising direction and five times in a decreasing direction. The median values are plotted to represent the pump performance curves are shown in Fig. 9. The CFD model is validated by comparing its outcomes to the tested data. The results of the CFD simulation predicts the pump’s performance for the baseline model. The validation outcomes demonstrate the CFD model’s capacity to forecast the performance of the designed model with 1% average deviation in Head and $<\,3\%$ in efficiency.

## Results

The operating conditions of the baseline model at the Best Efficiency Point (BEP) are chosen to be the input operating conditions to test the design tool. These operating conditions and all required assumption to start running the dsign tool are represented in Table 1. The design tool run the 1D model after specifying the input parameters. The 1D model solves the equations which are arranged in “One dimensional model” section and calculated the impeller main dimenssions and all the output parameters which are required for the next step (3D CFD model).

**Table 1:** Centrifugal pump input parameters and assumptions.

No.	Parameter	Symbol	Value	Units	Note
1	Head	H	5	(m)	(BEP) of the baseline model
2	Volumetric flow rate	Q	3.8	(${\text {m}}^3$/s)	
3	Rotational speed	n	1500	(rpm)	
4	Impeller type	Type	2	(–)	1. Closed (or) 2. Semi-open
5	Inlet flow angle	$\alpha _1$	90	(^∘^)	
6	Meridional velocity ratio	$c_{2m}/c_{1m}$	1	(–)	
7	Blade count	z	6	(–)	
8	Impeller entry count	$f_q$	1	(–)	1. Single (or) 2. Double
9	Hub to shaft diameter ratio	$d_w/d_n$	1.5	(–)	
10	Leading edge profile	$LE_p$	2	(–)	1. Semi-circle, 2. Elliptic (or) 3. Wedge
11	Trailing edge profile	$TE_p$	1	(–)	1. Tapered (or) 2. Full thickness

After specifying the required operating conditions and assumptions, the 1D model generates an output file that includes all the impeller’s geometrical data. The output file data is represented in Table 2.

**Table 2:** 1D model output.

No.	Parameter	Symbol	Value	Units	Note
1	Outlet diameter	$d_2$	134.8	(mm)	
2	Inlet diameter	$d_1$	50.8	(mm)	
3	Shaft diameter	$d_n$	10.5	(mm)	
4	LE inner diameter	$d_1i$	35	(mm)	At hub
5	LE outer diameter	$d_1a$	57.5	(mm)	At shroud
6	Impeller axial length	$Z_E$	27.5	(mm)	
7	Impeller side clearance	*s*	93.75	(%)	Percentage of span

**Figure 10:**
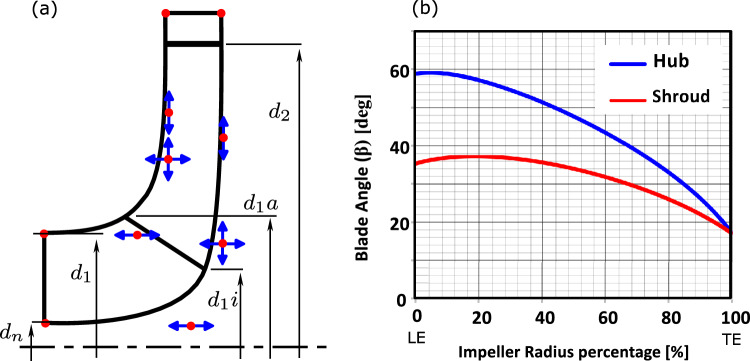
3D module input data (**a**) meridional contour (**b**) blade angle distribution.

**Figure 11:**
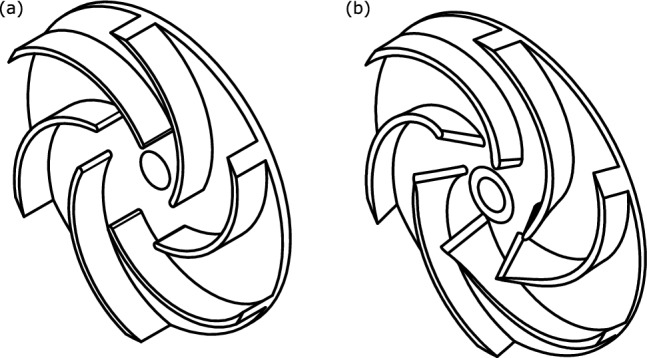
Impeller 3D CAD model: (**a**) baseline, (**b**) designed.

**Figure 12:**
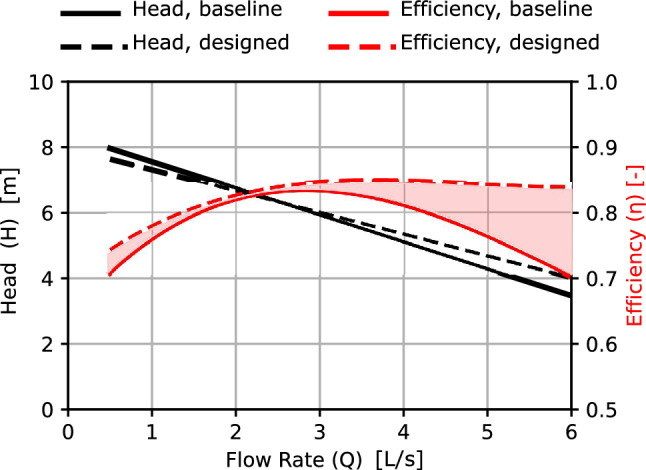
Impeller performance comparison, CFD.

The design tool’s 3D Module is launched after the 1D Module has been terminated. First, the 3D module builds the meridional contour based on the generated impeller geometries as shown in Fig. 10a. Second, a 3D model for the new impeller based on blade angles Fig. 10b and thickness distribution (3 mm constant) is created as shown in Fig. 11. Finally, the 3D module develops a CFD model to simulate pump operation and determine its performance as shown in Fig. 12.

The performance of the designed impeller is compared to the baseline model. The head curve of the designed impeller is almost identical to the baseline impeller head. The hydraulic efficiency of the designed impeller is higher than the baseline impeller. the rise in efficiency is increased with increasing the volumetric flow rate. Therefore, the design tool succeeded in designing the impeller and achieving the required head and flow rate with better hydraulic efficiency even before doing any optimization process.

To compare the performance of both the baseline and the designed impeller, the sources of the losses are evaluated by calculating the Entropy Generation Rate (EGR) in the simulated impellers. Recent studies^10–13^ have used this criterion to acquire the volumetric entropy generation rate by calculating the viscous dissipation $(S_v)$ and the thermal diffusion $(S_t)$ as shown in Eqs. (27) and (28), respectively.

27 $$\begin{aligned}{} & {} \begin{aligned} \dot{S_v}&= \frac{\mu _{eff}}{T} + \left\{ 2 \left[ \left( \frac{\partial u}{\partial x}\right) ^2 + \left( \frac{\partial v}{\partial y}\right) ^2 +\left( \frac{\partial w}{\partial z}\right) ^2 \right] \right. \\&\quad \left. + \left[ \left( \frac{\partial u}{\partial y}+\frac{\partial v}{\partial x}\right) ^2 + \left( \frac{\partial u}{\partial z} +\frac{\partial w}{\partial x} \right) ^2 +\left( \frac{\partial v}{\partial z} +\frac{\partial w}{\partial y} \right) ^2 \right] \right\} \end{aligned} \end{aligned}$$

28 $$\begin{aligned}{} & {} \dot{S_T} = \frac{\lambda _{eff}}{T^2} + \left[ \left( \frac{\partial T}{\partial x}\right) ^2 + \left( \frac{\partial T}{\partial y}\right) ^2 +\left( \frac{\partial T}{\partial z}\right) ^2 \right] \end{aligned}$$

The EGR from the thermal diffusion term is negligible in comparison to viscous dissipation in the case of centrifugal pump flow and could be disregarded^13–15^. The EGR might be decomposed into two portions, time-averaged ($\bar{\dot{S_v}}$) and fluctuating ($\tilde{{S_v}}$), as illustrated in Eqs. (29–30), respectively, based on the concept of Reynolds decomposition. After computing the time-averaged value of the velocity gradient, the time-averaged component ($\bar{\dot{S_v}}$) of the EGR may be assessed directly using any CFD solver. Equation (31) may be used to roughly represent the fluctuating portion of the EGR Eq. (30)^16,17^. Here, k and $\omega$ represent the SST $k-\omega$ model’s turbulent kinetic energy and characteristic frequency, respectively. According to the open literature, 0.09 is chosen for the empirical constant $\alpha$.

29 $$\begin{aligned}{} & {} \begin{aligned} \bar{\dot{S_v}}&= \frac{\mu _{eff}}{T} + \left\{ 2 \left[ \left( \frac{\partial {\bar{u}}}{\partial x}\right) ^2 + \left( \frac{\partial {\bar{v}}}{\partial y}\right) ^2 +\left( \frac{\partial {\bar{w}}}{\partial z}\right) ^2 \right] \right. \\&\quad \left. + \left[ \left( \frac{\partial {\bar{u}}}{\partial y}+\frac{\partial {\bar{v}}}{\partial x}\right) ^2 + \left( \frac{\partial {\bar{u}}}{\partial z} +\frac{\partial {\bar{w}}}{\partial x} \right) ^2 +\left( \frac{\partial {\bar{v}}}{\partial z} +\frac{\partial {\bar{w}}}{\partial y} \right) ^2 \right] \right\} \end{aligned} \end{aligned}$$

30 $$\begin{aligned}{} & {} \begin{aligned} \tilde{{S_v}}&= \frac{\mu _{eff}}{T} + \left\{ 2 \left[ \left( \frac{\partial {\tilde{u}}}{\partial x}\right) ^2 + \left( \frac{\partial {\tilde{v}}}{\partial y}\right) ^2 +\left( \frac{\partial {\tilde{w}}}{\partial z}\right) ^2 \right] \right. \\&\quad \left. + \left[ \left( \frac{\partial {\tilde{u}}}{\partial y}+\frac{\partial {\tilde{v}}}{\partial x}\right) ^2 + \left( \frac{\partial {\tilde{u}}}{\partial z} +\frac{\partial {\tilde{w}}}{\partial x} \right) ^2 +\left( \frac{\partial {\tilde{v}}}{\partial z} +\frac{\partial {\tilde{w}}}{\partial y} \right) ^2 \right] \right\} \end{aligned} \end{aligned}$$

31 $$\begin{aligned}{} & {} \tilde{{S_v}} = \alpha \frac{\rho \omega k}{T} \end{aligned}$$

**Figure 13:**
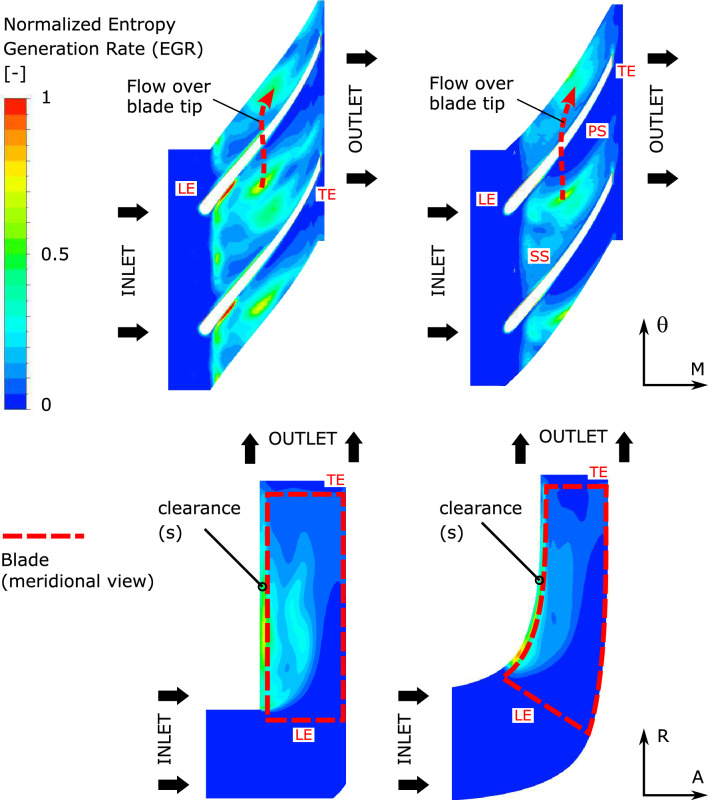
Entropy generation rate contours, top: stream-wise direction (50% span) bottom: meridional direction, Q = 5 L/s.

After calculating the EGR in the CFD domains, the EGR contours for the baseline and designed impeller are plotted as illustrated in Fig. 13. these contours prove how the designed impeller achieves lower shock losses at the over-load operating region compared to the simple baseline model. The EGR at $Q=5 L/s$ has been chosen to be plotted because it makes it easier to analyze the efficiency and losses due to the wide difference for both cases (base line and designed) at this operating point.

## Conclusions

A design tool is developed to design the impeller of the centrifugal pump. It integrates 1D and CFD models to benefit from the computing power and extensive knowledge in this field. The study’s findings indicate that:

- Choosing the right design factors and integrating the 1D and CFD models results in an effective centrifugal impeller selection before applying any optimization algorithms as shown by the proposed design tool.
- Monitoring the entropy generation rate during the design process allows the design tool to quantify the hydraulic and volumetric losses in the impeller and assess the designed impeller.
- The centrifugal impeller may be optimized for the chosen operating point using an optimization routine that is built around the tool that has been described.

## Data Availability

The datasets used during the current study are available from the corresponding author upon reasonable request.

## List of symbols

*A*: Area ($${\text {m}}^2$$)
*b*: Impeller width (m)
*c*: Absolute velocity (m/s)
*d*: Impeller diameter (m)
*e*: Blade thickness (m)
$f_q$: Impeller entry count (–)
*H*: Pump head (m)
*Q*: Pump volumetric flowrate (${\text {m}}^3/{\text {s}}$)
*n*: Rotational speed (rpm)
$n_q$: Specific speed (rpm)
*P*: Power (W)
*r*: radius (m)
*s*: Impeller side clearance (m)
${\dot{S}}$: Entropy generation rate (${\text {J\,K}}^{-1}{\text {s}}^{-1}$)
*u*: Circumferential velocity (m/s)
*w*: relative velocity (m/s)
*z*: Blade count (–)
$Z_E$: impeller axial extension (m)
$$\alpha $$: Flow angle (^∘^)
$$\beta $$: Blade angle (^∘^)
$\lambda$: Angle between vanes and side disks (^∘^)
$$\phi $$: Flow coefficient (–)
$$\eta $$: Efficiency (–)
$\tau$: Impeller blockage factor due to blade thickness (–)
$$\psi $$: Head coefficient (–)

## Subscripts

1: Impeller inlet (at shroud)
1*a*: Blade LE (at shroud)
2: Impeller outlet
*al*: Allowable
*B*: Blade
*h*: Hydraulic
*La*: Impeller–rotor
*Le*: Volute-casing
*m*: Mechanical
*m*: Meridional direction
*max*: Maximum
*n*: Impeller inlet at hub
*opt*: operation at maximum (best) efficiency (BEP)
*Ref*: Reference
*v*: Volumetric
*w*: Shaft

## References

[1] Insights, F. B. *Centrifugal pump market report*. Website https://www.fortunebusinessinsights.com/industry-reports/centrifugal-pump-market-101079 (2022).

[2] Augustyn, T. Energy efficiency and savings in pumping systems-the holistic approach. In *2012 Southern African Energy Efficiency Convention (SAEEC)* 1–7. 10.1109/SAEEC.2012.6408587 (2012).

[3] Shankar VKA, Umashankar S, Paramasivam S, Hanigovszki N (2016). A comprehensive review on energy efficiency enhancement initiatives in centrifugal pumping system. Appl. Energy.

[4] Capurso T, Bergamini L, Torresi M (2022). A new generation of centrifugal pumps for high conversion efficiency. Energy Convers. Manag..

[5] Hassan AFA, Abdalla H, Aly AAE-A (2017). Centrifugal pump performance enhancement by blade shape modification. Turbo Expo Power Land Sea Air.

[6] Mousmoulis G, Kassanos I, Aggidis G, Anagnostopoulos I (2021). Numerical simulation of the performance of a centrifugal pump with a semi-open impeller under normal and cavitating conditions. Appl. Math. Model..

[7] Gülich, J. F. *Operation Of Centrifugal Pumps* (Elsevier, 2008).

[8] Hassan, A. F. S. A. *Experimental and Computational Study of Semi-open Centrifugal Pump*. Master Thesis, Military Technical College, Cairo, Egypt. 10.13140/RG.2.1.5190.3766 (2015).

[9] Ayad AF, Abdalla HM, Aly AAE-A (2015). Effect of semi-open impeller side clearance on the centrifugal pump performance using cfd. Aerosp. Sci. Technol..

[10] Iandoli, C. *Analysis of the Entropy Generation Fields of a Low ns Centrifugal Compressor*, vol. 1 (ME Thesis, University of Roma, 2000).

[11] Iandoli CL, Sciubba E (2000). Entropy generation maps of a low-specific speed radial compressor rotor. ASME Int. Mech. Eng. Cong. Expos..

[12] Sciubba E (2005). Computing the entropy generation rate for turbomachinery design applications: Can a diagnostic tool become a predictive one?. ASME Int. Mech. Eng. Cong. Expos..

[13] Hassan, A. F. A., Schatz, M. & Vogt, D. M. Performance and losses analysis for radial turbine featuring a multi-channel casing design. *J. Turbomach.*10.1115/1.4049611 (2021).

[14] Copeland CD, Newton PJ, Martinez-Botas R, Seiler M (2011). The effect of unequal admission on the performance and loss generation in a double-entry turbocharger turbine. J. Turbomach..

[15] Newton P, Copeland C, Martinez-Botas R, Seiler M (2012). An audit of aerodynamic loss in a double entry turbine under full and partial admission. Int. J. Heat Fluid Flow.

[16] Kock F, Herwig H (2004). Local entropy production in turbulent shear flows: A high-Reynolds number model with wall functions. Int. J. Heat Mass Transf..

[17] Li X, Zhu Z, Li Y, Chen X (2016). Experimental and numerical investigations of head-flow curve instability of a single-stage centrifugal pump with volute casing. Proc. Inst. Mech. Eng. A J. Power Energy.

